# Bio-Nanohybrid Gelatin/Quantum Dots for Cellular Imaging and Biosensing Applications

**DOI:** 10.3390/ijms231911867

**Published:** 2022-10-06

**Authors:** Sangram Keshari Samal, Stefaan Soenen, Dario Puppi, Karolien De Wael, Sanghamitra Pati, Stefaan De Smedt, Kevin Braeckmans, Peter Dubruel

**Affiliations:** 1 Laboratory of Biomaterials and Regenerative Medicine for Advanced Therapies, Indian Council of Medical Research-Regional Medical Research Center, Bhubaneswar 751013, Odisha, India; 2 Laboratory of General Biochemistry and Physical Pharmacy, Center for Nano- and Biophotonics, Ghent University, Ottergemsesteenweg 460, 9000 Ghent, Belgium; 3 Polymer Chemistry & Biomaterials Research Group, Ghent University, Krijgslaan 281-S4, 9000 Gent, Belgium; 4 Biomedical MRI Unit/MoSAIC, KU Leuven Department of Medicine, Herestraat 49, 3000 Leuven, Belgium; 5 BioLab Research Group, Department of Chemistry and Industrial Chemistry, University of Pisa, UdR INSTM-Pisa, Via Moruzzi 13, 56124 Pisa, Italy; 6 Department of Chemistry, University of Antwerp, Universiteitsplein 1, 2610 Antwerp, Belgium

**Keywords:** gelatin, quantum dots, biocompatible, cellular imaging, biosensor

## Abstract

The bio-nanohybrid gelatin protein/cadmium sulfide (Gel/CdS) quantum dots (QDs) have been designed via a facile one-pot strategy. The amino acids group of gelatin chelate Cd^2+^ and grow CdS QDs without any agglomeration. The ^1^H NMR spectra indicate that during the above process there are no alterations of the gelatin protein structure conformation and chemical functionalities. The prepared Gel/CdS QDs were characterized and their potential as a system for cellular imaging and the electrochemical sensor for hydrogen peroxide (H_2_O_2_) detection applications were investigated. The obtained results demonstrate that the developed Gel/CdS QDs system could offer a simple and convenient operating strategy both for the class of contrast agents for cell labeling and electrochemical sensors purposes.

## 1. Introduction

Cellular imaging and biosensing technology have gained great interest in various therapeutic applications including cell imaging, biosensor, diagnostic and drug delivery systems [1,2,3,4,5,6]. In recent years, several inventive designs for various cellular imaging and sensor biomaterials have been developed. Biocompatible Quantum Dots (QDs) are a new class of fluorescently labeled biomaterials with considerable recent interest for various diagnosis and therapeutic applications [7,8]. These materials have excellent optical and electronic properties compared to organic dyes and fluorescent protein, with size-tunable light emission, resistance to photobleaching and superior signal brightness, which make them appealing for application in the field of sensor and cellular imaging applications [2]. Similarly, QDs-based electrochemical assay is an emerging scientific topic. Among various types of QDs, Cadmium Sulfide (CdS) QDs have been extensively studied due to their possible applications in several technological areas, such as chemical or biological sensors, gene delivery and cellular imaging applications [9,10,11,12]. 

The drawback of the QDs arises from their loss in photoluminescence property due to oxidation or environmental biomolecule interaction during transportation, long-term storage and physiological environment use for bio-applications. The use of QDs for biological applications in a physiological environment interacts with the present biomolecules, salts and buffers, resulting in QDs cluster formation or precipitation. These phenomena induce negative impacts on the photoluminescence properties of QDs, which is a major drawback for its utilization. Hence, to address the above-mentioned limitations and to retain QDs photoluminescence stability, the present study has focused on developing a bio-nanohybrid system based on gelatin and semiconductor QDs. Gelatin is a biopolymer derived from collagen consisting of glycine, proline, 4-hydroxyproline and triple helix [13]. Gelatin solution acts as a good dispersion medium for nano-biomaterials, as metal and chalcogen ions easily penetrate into the molecular chain of gelatin [14,15]. Furthermore, gelatin-based nano-biomaterials have demonstrated excellent biocompatibility of the immobilized enzyme and its activity for sensor applications [16]. The hydrophilic domains of gelatin provide a biomimetic microenvironment for proteins, enzymes, cells and other natural substances with maintaining their native configuration, which makes gelatin a suitable matrix for small molecule entrapment. Several enzymes have been incorporated so far into a gelatin matrix, in order to develop an electrochemical biosensor [17]. Embedding more than one enzyme in gelatin has also been explored as a suitable strategy for biosensor fabrication [18]. Nanotechnology amplification processes have enhanced the intensity of the imaging signal and can lead to ultrasensitive assays [19,20]. The combination of biopolymer and nanotechnology provides inherent miniaturization, high sensitivity and is cost-effective for sensor and imaging technology [21,22]. The nanohybrid system developed in the present study was aimed to be used as an effective sensor for H_2_O_2_ detection. H_2_O_2_ is a simple compound used as an effective oxidant. It is a necessary component for the metabolism of carbohydrates, vitamins and proteins and plays a crucial role in regulating molecular signaling in biological systems [23,24]. In living organisms, oxidative damages in the human body are caused due to the cellular imbalance of H_2_O_2_, which is an important component for cell signaling and communication. It acts as a mediator in biology and medicine and is a byproduct generated during biochemical enzymatic oxidation of several highly selective oxidases [25]. Hence, it is essential to have accurate and sensitive detection of H_2_O_2_ [26]. There are several conventional analytical techniques employed for the detection of H_2_O_2_ such as spectrophotometry, colorimetry and titrimetric, which are complex, expensive and time-consuming. However, the electrochemical method offers an easy, sensitive and cost-effective means to detect electroactive H_2_O_2_ [27].

**Figure 1:**
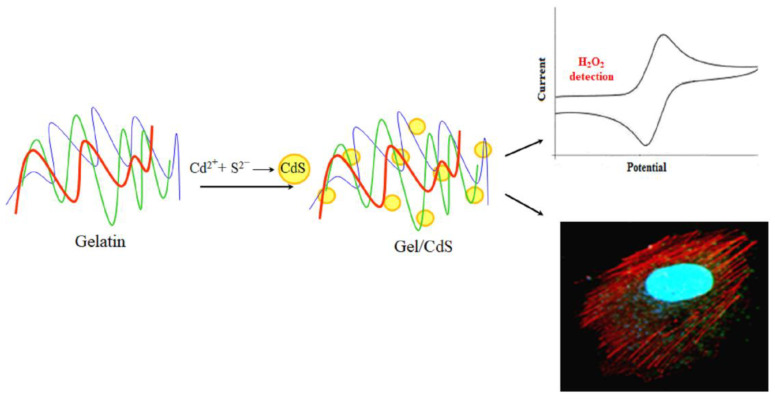
Biomimetic synthesis of cadmium sulfide (CdS) QDs in a biopolymer gelatin template as a system for biosensor and cellular imaging applications.

In the present study, a facile single-step synthesis strategy was demonstrated to generate the hybrid nanostructures, Gel/CdS QDs. This system can act as an effective carrier to provide sufficient fluorescent stability of CdS QDs over a longer period of time and explore potential applications both in biosensing and cellular imaging. The stability of this Gel/CdS QDs system is due to the chelation of gelatin’s amine group with cadmium metal ion. The most attractive property of the developed system is its long storage potential without losing its efficacy. We report on the electrochemical sensing behavior of Gel/CdS QDs on a gold electrode surface for application in H_2_O_2_ detection and cellular imaging potential of Human Umbilical Vein Endothelial Cells (HUVEC) (Figure 1).

## 2. Results and Discussion

The Gel/CdS hybrid nanostructure system presented in this work is constituted by CdS QDs incorporated in a gelatin matrix. The synthesis procedure of CdS nanocrystals involves one step, in which the aqueous gelatin solution, Cd(NO_3_)_2_ and Na_2_S, dissociates into Cd^2+^ and S^2−^ ions, respectively. The S^2−^ has a molecular volume of about 25 A^°^ which leads it to penetrate easily into the gelatin molecular chain, wherein Cd^2+^ are chelated by amino and carboxyl groups of the amino acid residues, resulting in a consequent growth of CdS QDs. Resistance to larger growth of CdS QDs and further agglomeration is provided by the gelatin molecular chains. The major advantage of the above synthesis process is the one-pot, one-step synthesis procedure that avoids coagulation of the CdS QDs and hinders their further growth. This system can be stored for a long period of time and it can be then resuspended for further experiments without losing its photoluminescence properties. This specific property is important for cell labeling, which makes this Gel/CdS QDs system unique and interesting.

To summarize, the Gel/CdS QDs were prepared through an exchange chemical reaction occurring in the gelatin solution following the reaction,

Gelatin + Cd(NO_3_)_2_ + Na_2_S → Gel/CdS + 2NaNO_3_ (1)

**Figure 2:**
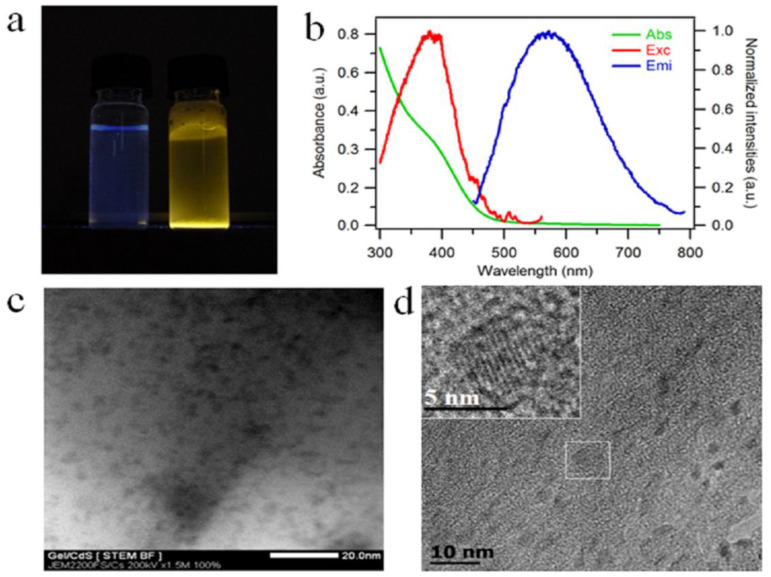
(**a**) Gelatin solution (left) and Gel/CdS QDs (right) under UV light (λ_ex_ = 365 nm), (**b**) optical analysis of CdS QDs growth on gelatin aqueous solution, absorption spectrum (green line, left Y axis), excitation and emission spectra (solid lines red and blue, right Y axis), (**c**) STEM and (**d**) TEM images, inset enlargement on one particle showing lattice fringes.

With the progress of the reaction, the growth of the CdS QDs leads to a color change of the gelatin solution from blue to yellow under UV light (Figure 2a). This color change can be attributed to the quantum confinement of the CdS QDs.

The presence of CdS nanocrystals in the gelatin matrix is evaluated using luminescence spectroscopy and optical absorption. The UV-visible absorption and photoluminescence spectra (excitation and emission) are reported in Figure 2b. From the representative absorption spectrum, a shoulder is visible at 390 nm in spite of the scattering of light by the sample at shorter wavelengths. The position of this absorption peak is confirmed by the photoluminescence spectra where we observe a maximum in the excitation spectrum at the same wavelength. The broad emission centered at 600 nm is characteristic of trap-state emission. This trap-state emission is related to surface defects of the nanocrystals due to gelatin protein and has already been reported for CdS quantum dots prepared under similar conditions using DNA as a template [10]. The transmission electron microscopy (TEM) and scanning transmission electron microscopy (STEM) images of Gel/CdS QDs reveal nanoparticles within the gelatin matrix (Figure 2c,d) [28]. Elemental analysis of these particles with TEM-based energy-dispersive X-ray analysis (TEM-EDS) and Scanning Electron Microscope-EDS confirmed the presence of Cd and S (Figures S1 and S2). These CdS QDs exhibit an oblate shape [10], with sizes of CdS QDs of average length at 4.4 ± 0.7 nm along the long dimension) and the average length along the short dimension is 2.1 ± 0.5 nm (short dimension), i.e., an aspect ratio of 2.1. The gelatin molecular chains effectively prevent the agglomeration of the CdS particles. As it is visible in the inset of Figure 2d, CdS QDs exhibit lattice fringes highlighting their crystalline character.

**Figure 3:**
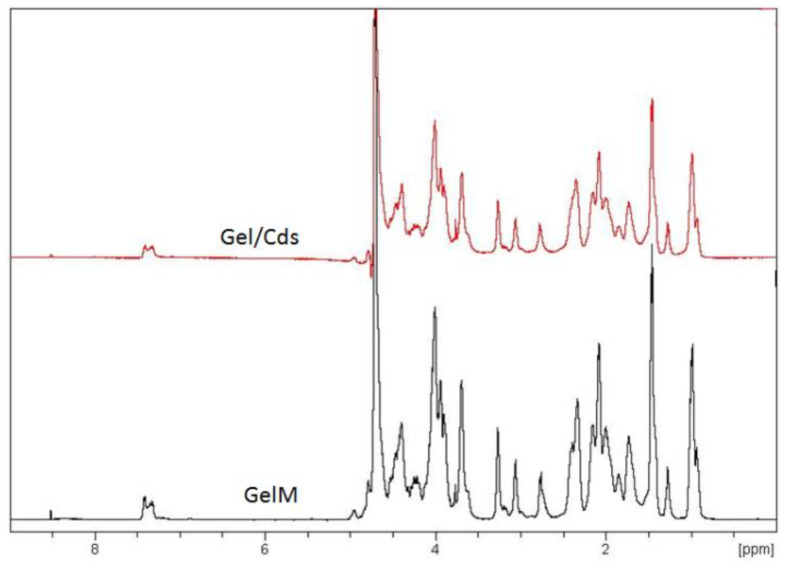
^1^H-NMR spectrum of gelatin and Gel/CdS.

The ^1^H NMR spectroscopy technique was used to investigate the interaction and mobility of the molecular chain of gelatin. The ^1^H-NMR spectrum of gelatin and Gel/CdS QDs at 40 °C is shown in Figure 3. Most of the proton signals of the gelatin are well resolved and assigned to specific amino acids [29,30,31,32,33]. The result showed that there were no different peaks in Gel/CdS spectra, which indicate there were no chemical modification in this system. An important observation that was made from this analysis is that gelatin as an organic matrix is not affected by the synthesis procedure of CdS QDs and its benefits can still be retained.

**Figure 4:**
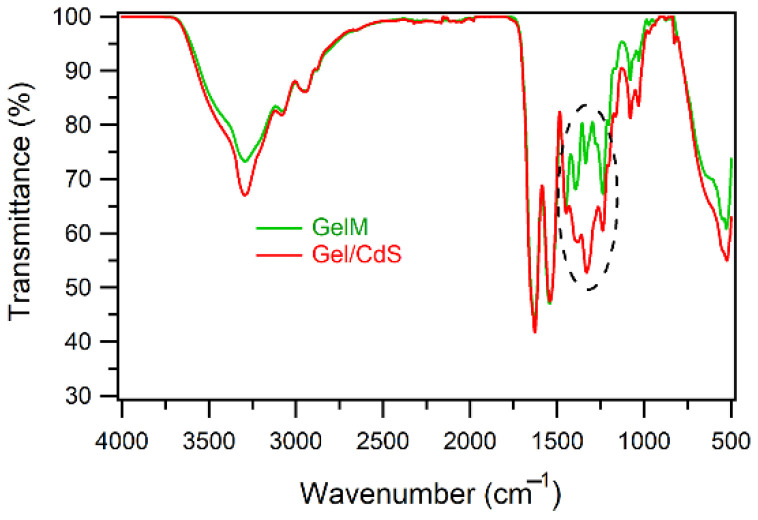
ATR-FTIR spectra of gelatin and Gel/CdS QDs.

The ATR-IR spectrum (Figure 4) of the gelatin membrane (GelM) shows characteristic regions namely, amide A, amide I, amide II and amide III bands. The amide A mode consists of bands at 3324, 2935 and 2837 cm^−1^ that correspond to NH stretch, hydrogen bonding, and CH_2_ symmetrical and asymmetrical stretch, respectively. Furthermore, the amide I characteristic band observed at 1653 cm^−1^ is due to the triple helices and a band at 1638 cm^−1^ which is for random coils. The amide II region bands at 1550 cm^−1^ due to NH bend coupled with CN stretch, 1450 cm^−1^ because of CH_2_ bending and 1338 cm^−1^ for CH_2_ of proline. The characteristic region of amide III is composed of three characteristic bands. The band at 1168 cm^−1^ corresponds to an NH bending, the C–O stretch is responsible for the band at 1083 cm^−1^ and the band at 1034 cm^−1^ is related to skeletal stretches. The Gel/CdS QDs also showed a similar pattern as that of the pure gelatin with some changes in the region 1200–1500 cm^−1^. The changes might be due to inorganic CdS.

**Figure 5:**
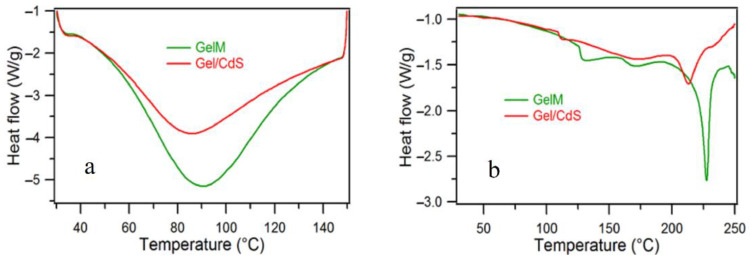
DSC spectra of (**a**) 1st heating and (**b**) 2nd heating of gelatin and Gel/CdS QDs.

The DSC thermogram indicated the difference between the two investigated samples. The 1st heating scan (Figure 5a) shows gelatin as more hydrophilic compared to Gel/CdS QDs, in other words, the CdS QDs decreases the hydrophilicity of gelatin. The presence of the nanostructured CdS QDs helps faster evaporation of water which is evident from the shift of the relevant endothermic peak in the thermogram. The 2nd heating scan (Figure 5b) reveals a clear change in the degradation temperature of Gel/CdS QDs in comparison to GelM. This could be due to the presence of CdS which increases the thermal conductivity of the gelatin matrix, thereby degrading faster than pure gelatin.

**Figure 6:**
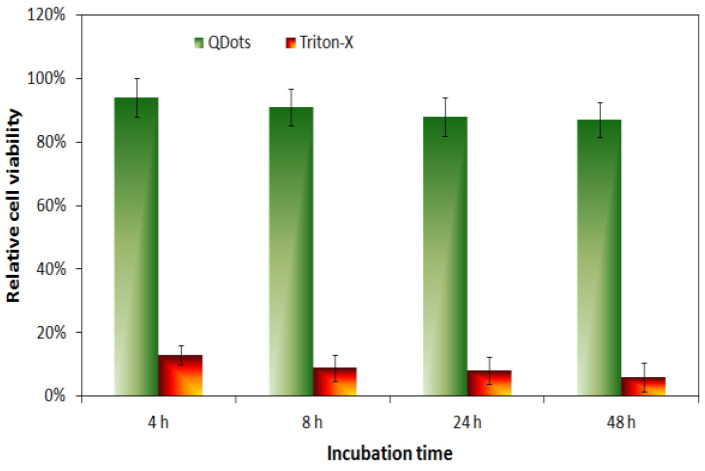
Relative cell viability of HUVEC cells exposed to Gel/CdS QDs at 5 µg/mL for 4, 8, 24 or 48 h as assessed through an Alamar blue assay.

The cellular imaging potential of the Gel/CdS QDs was investigated using HUVEC and their effects on toxicity and cellular uptake were assessed. Initially, HUVEC cells were exposed to a concentration series of Gel/CdS ranging from 0–5 µg/mL. Under these conditions, no acute cytotoxic effects were observed using either a lactate dehydrogenase or an Alamar blue assay. The data from the Alamar blue assay further showed no effect of the particles on the proliferative capacity of the cells which was confirmed by manual cell counting studies at later time points (Figure 6).

The obtained results confirmed that up to a concentration of 5 µg/mL Gel/CdS do not appear to have any immediate cytotoxic effects on the HUVEC cells. The lack of toxicity seen here due to the presence of the gelatin matrix can possibly hint at a biocompatible coating of the particles [34,35,36,37]. This result is in line with a previous report by Byrne and colleagues, where the toxicity of small ligand-coated QDs could be reduced by adding a second gelatin-based coating on top of the primary one [38]. Alternatively, the gelatin coating may result in a large reduction in cellular uptake as most toxic effects are linked with the intracellular presence of the QDs (e.g., induction of reactive oxygen species or leaching of toxic Cd^2+^ ions upon intraendosomal degradation) [39,40]. As the QDs are to be used for cell labeling purposes, it is pertinent that they are efficiently taken up by the cells and enable clear fluorescent cell visualization. The uptake of the Gel/CdS QDs by HUVEC was evaluated by confocal laser microscopy after exposing the cells to the particles for 24 h at 5 µg/mL.

**Figure 7:**
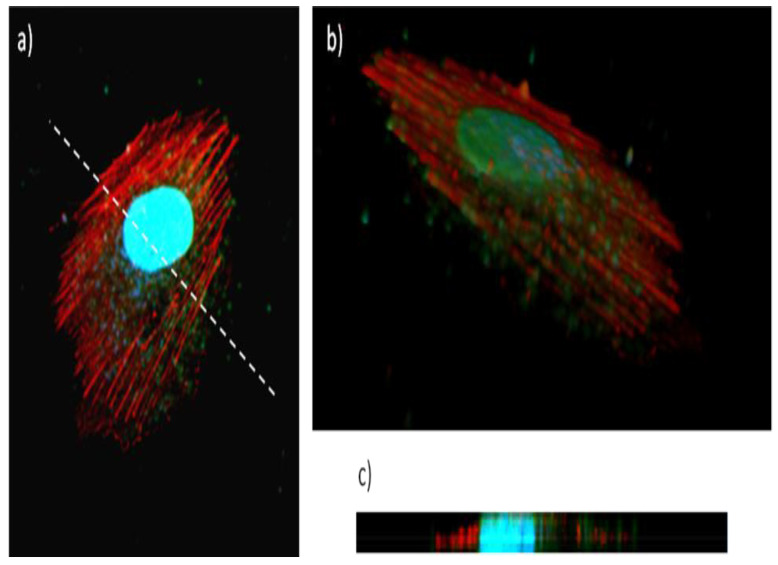
Uptake of Gel/CdS QDs (green) by HUVEC exposed for 24 h at 5 µg/mL, after which the cells were stained for actin (red) and subjected to nuclear counterstaining (blue), (**a**) shows a confocal slice in the middle of the cell (slice 7) revealing high levels of QDs within the cellular environment, between the actin fibers, (**b**) shows a 3D view of a labelled cell, consisting of a composition of 15 slices with 0.24 µm interslice distance, (**c**) shows a projection along the Z-axis of the 3D figure, taken along the line drawn in (**a**). These data reveal high levels of Gel/CdS QDs internalized by the cells.

Figure 7 clearly shows high levels of cell-associated Gel/QDs, which result in a punctuate staining pattern throughout the entire cytoplasm but is the most pronounced in the perinuclear region. This type of staining is indicative of endosomal localization of the Gel/CdS QDs, which is in line with previous reports on nanoparticles [40,41]. As the image selected is a confocal slice from a HUVEC cell taken at medium height, the high presence of particles between the actin fibers indicates high levels of internalized particles. A 3D reconstruction of the cell with the cell nuclei and actin filaments stained (Figure 7b,c) confirms this data, showing the clear intracellular presence of high levels of the Gel/CdS QDs. Previous studies on CdS QDs of similar size but without a cellular coating found much higher levels of toxicity, where the IC_50_ value was 4 µg/Ml [42]. The data obtained here suggest that the gelatin coating significantly impairs CdS toxicity, while the high level of uptake and efficient fluorescent properties suggest that these particles are well-suited as tools for fluorescence microscopy and as contrast agents for cell labelling purposes.

**Figure 8:**
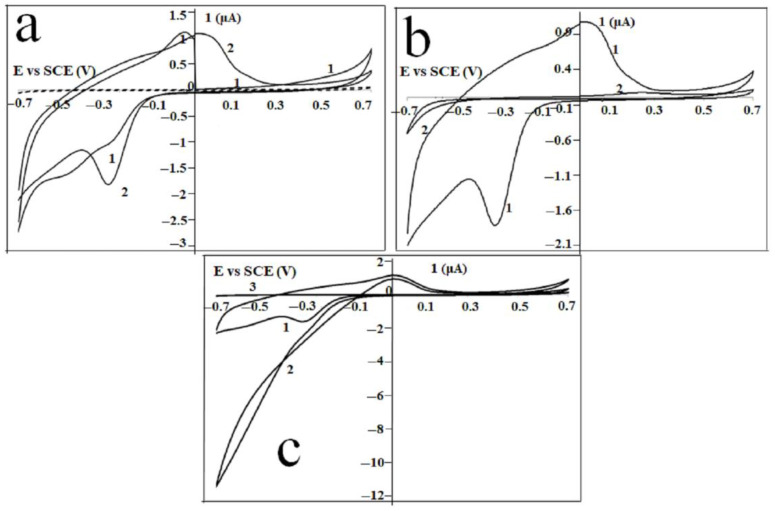
Current-potential behavior of (**a**) Au|Gel (dotted) and Au|Gel/CdS electrode (solid) in HEPES buffer solution: first (1) and fifth (2) scan, and (**b**) Au|Gel/CdS (1) and a Au|MOH|Gel/CdS (2) electrode in a HEPES buffer solution. Scan 5 of each experiment is shown, (**c**) current-potential behavior of Au|Gel/CdS electrode in the absence (1) and presence (2) of mM H_2_O_2_. Curve 3 is the current-potential behavior of Au|Gel electrode in presence of mM H_2_O_2_.

The electrochemical behavior of Gel/CdS QDs immobilized at a gold electrode was observed by the cyclic voltammetric analysis of Au|Gel/CdS electrode in the HEPES buffer solution represented in Figure 8. The first and fifth scans are presented. Compared to the dotted curve obtained at a bare Au/Gel electrode, not containing CdS QDs, a well-defined oxidation and reduction peak at, respectively, 0.02 V and −0.34 V (fifth scan) was observed and explained by the following reaction

Gel|Cd^2+^ + 2e^−^→ Gel|Cd (2)

At the beginning of the first scan, the oxidation peak is absent, which can be explained by the fact that no Cd is present when the cyclic voltammogram is initiated at 0 V. As soon as the potential is directed towards more negative potentials (ca −0.34 V) and Cd is formed, the re-oxidation process appears in the next sweep of the scan. As previously reported, when CdS QDs were formed in situ in gelatin, CdS QDs as well as Cd^2+^ ions, are both present in the hydrated gelatin matrix. Only the Cd^2+^ ions give rise to the typical electrochemical behavior as shown in Figure 8a,b.

The prominent set of peaks was observed at more positive values for the potential than the reversible Nernst potential for bulk deposition, i.e., −0.65 V vs SCE [43,44,45,46,47,48,49]. This direct detection of Cd^2+^ via the UPD approach has also been demonstrated previously using peptides as the matrix material [50,51]. Figure 8 clearly shows that when a mercaptohexanol (MOH) linker was used as a self-assembled monolayer, the UPD is prevented by the presence of the monolayer. Only a small fraction of the Cd^2+^ ions present in the gelatin matrix will be oxidized when a Au/MOH/Gel/CdS electrode is cycled in a HEPES buffer solution. This oxidation process starts at a more positive potential compared to the cyclic voltammetric behavior obtained at a Au|Gel/CdS electrode. The effect of MOH in H_2_O_2_ detection was investigated by using Au|MOH|Gel/CdS electrode. The cyclic voltammetric behavior of a Au|Gel/CdS electrode in the presence and absence of mM H_2_O_2_ are shown in Figure 8a,b. An increase in the reduction current was observed when H_2_O_2_ was added to the buffer solution. The onset of this catalytic process was observed at the UPD potential. To make a comparison with the electrochemical behavior of H_2_O_2_ at the Au|Gel electrode, curve 3 was added to Figure 8c. The bare electrode (not containing CdS QDs) curve shows no increased reduction current when H_2_O_2_ is added to the cell solution. These results show that the CdS QDs in the gelatin matrix catalyze redox reactions.

## 3. Conclusions

In summary, the present research describes a low-cost and easy one-step synthesis strategy for fabricating the Gel/CdS QDs membrane. The Gel/CdS does not alter the protein conformation and chemical functionality. This intercalation of CdS QDs into the protein makes them stable and robust to use. The cellular imaging result indicates a high level of uptake and efficient imaging potential without having any toxic effects. These Gel/CdS QDs are biocompatible and possess long storage life when processed as films/membranes. These films can be resuspended later for further use without losing their photoluminescence properties. The presence of CdS QDs within the gelatin matrix may be responsible for catalyzing redox reactions. These complementary characteristics make Gel/CdS QDs a unique and exciting system with a longer storage potential, a simple and convenient operating strategy to develop a new class of contrast agents for cellular imaging and electrochemical sensor applications.

## 4. Experimental Section

### 4.1. Materials

Cadmium nitrate (CdNO_3_. 4 H_2_O, 98%), sodium sulfide (Na_2_S), 2-[4-(2-hydroxyethyl)- piperazinyl] Ethane Sulfonic acid (HEPES), Sodium hydroxide and Mercapto Hexanol (MH) were purchased from Aldrich. Gelatin (type B) is isolated from bovine skin by an alkaline process. Gelatin possessing an Isoelectric point of around 5 and a Bloom strength of 257 was used. Milli-Q water was used for all experimental processes.

### 4.2. Preparation of Gelatin/Quantum Dots

In this study, a 3 mg/mL gelatin solution was prepared at 40 °C and Cd (NO_3_)_2_ was added. Other gelatin concentrations were also tried and 3 mg/mL was found to be the best formulation for luminescence property. Equimolar ratios of the cadmium and sulfur salts were used. After 15 min of mixing at 40 °C, 10 mL of Na_2_S solution was added dropwise with continuous stirring at 500 rpm. As the reaction began, the gelatin solution gradually changed from transparent to pale yellow and after the completion of the reaction, the gelatin solution turned to dark yellow. The Gel/CdS nanocrystals were purified by centrifugation and used for further analysis.

### 4.3. Photoluminescence Property of Gelatin/Quantum Dots

The optical properties of the Gel/CdS nanocrystals were analyzed using UV and photoluminescence spectroscopy. The UV absorption spectrum was recorded on a Perkin Elmer Lambda 950 spectrophotometer. The excitation and emission spectra were recorded using a quartz cuvette on Varian Cary Eclipse Fluorescence Spectrophotometer. The intensities of the excitation and emission spectra were normalized and corrected according to the sensitivity of the detector.

### 4.4. Gel/CdS Particle Morphology and Elemental Analysis

STEM, TEM and TEM-EDS analyses were performed by the JEM-2200FS FEG (JEOL) microscope instrument operated at 200 kV and equipped with a spherical aberration corrector. For morphology and EDS observations, a small piece of the film was placed between two copper grids and mounted on the sample holder. The ^1^H-NMR spectra of GelM and Gel/CdS were recorded at 40 °C in D_2_O by using a Bruker WH 500 MHz instrument and Topspin software. The chemical shift was expressed in ppm as a function of tetramethylsilane as the internal standard. This spectrum shows the different protons from amino acids of gelatin.

### 4.5. H NMR

Gelatin and Gel/CdS were dissolved in milli-Q water at a concentration of 10 mg/mL. ^1^H-NMR spectra of Gel and Gel/CdS were recorded at a temperature of 40 °C using a Bruker WH 500 MHz instrument and Topspin software. The chemical shift of ^1^H-NMR was expressed in ppm with the function of tetramethylsilane as an internal standard. This spectrum shows the different protons from amino acids of gelatin.

### 4.6. ATR-FTIR Measurement

ATR-FTIR measurements were performed by using a Biorad FT-IR spectrometer FTS575C (Bio-Rad, Nazareth, Belgium), which is equipped with a “Golden Gate” ATR accessory (Specac, Kent, UK) and fitted with a diamond crystal. The ATR-FTIR spectra were taken as an average of 32 scans with 4 cm^−1^ of resolution in the region of 4000 to 500 cm^−1^ using WIN-IR software.

### 4.7. Differential Scanning Calorimeter (DSC)

Thermal properties of the gelatin/CdS QDs were characterized by differential scanning calorimeter (DSC) from TA, model DSC822 with TA software. The sample size was 3–5 mg weighed in a standard 40 μL aluminum pan and an empty pan was used as reference. The temperature of the DSC instrument was calibrated using indium, lead and zinc standards and energy calibration using indium standards.

The DSC measurements were performed in accordance with the ASTM D 3418 standard method under a helium gas flow rate of 80 mL·min^−1^. The detailed methods for the DSC characterization according to the following protocol:

1. First heating scan range from 30 °C to 150 °C at 10 °C·min^−1^ and 2 min of isotherm at the end;
2. First cooling scan from 150 °C to −25 °C at 10 °C·min^−1^ and 2 min of isotherm at the end;
3. Second heating scan from −25 °C to 250 °C at 10 °C·min^−1^.

### 4.8. Cellular Exposure

The cellular imaging potential of the Gel/CdS QDs was investigated using HUVEC. The HUVEC cells were obtained from Lonza (Belgium) and kept in a basal endothelial medium with a supplement (Gibco, Belgium). For uptake studies, HUVECs were seeded at 2 × 10^4^ cells/dish in collagen-coated glass-bottom confocal dishes (MatTek, USA) and allowed to settle overnight. Then, cells were exposed to the Gel/CdS QDs for 24 h at 5 µg/mL. Following incubation, the medium was aspirated, cells were washed twice with PBS and fixated in 4% paraformaldehyde for 10 min at ambient temperature. Next, cells were permeabilized for 10 min by 0.1% Triton X-100 in PBS followed by incubation with 10% serum-containing PBS for 30 min. Then, cells were incubated with Alexa Fluor 555-conjugated Phalloidin (Molecular Probes, Invitrogen, Belgium) at 1/300 dilution in 10% serum-containing PBS for 1 h at ambient temperature. This medium was then aspirated, cells were washed twice with PBS after which they were counterstained with DAPI for 10 min prior to visualization by a Nikon C1 confocal microscope. The toxicity of the Gel/CdS QDs was evaluated by a standard Alamar Blue assay (Molecular Probes, Belgium). In brief, HUVEC cells were seeded at 2 × 10^4^ cells/well in 96 well plates after which the cells were allowed to settle overnight. Then, cells were incubated with the Gel/CdS QDs at 0, 1, 2, 3, 4, 5 µg/mL and the Alamar Blue assay was performed according to the manufacturer’s instructions after 4, 8, 24 and 48 h, and the kinetic readouts were performed on the same plate using a FluoStar Optima plate reader (BMG LabTech, UK). These assays were performed in triplicate.

### 4.9. Electrode Preparation

Electrochemical experiments were performed with reference to the previously published protocol. Briefly, in the current experimental set-up, a three-electrode cell using a saturated calomel reference electrode (SCE) containing two compartments (Radiometer Analytical, Lyon, France) and a platinum counter electrode were used. The working electrodes were gold electrodes with a diameter of 1.6 mm (BAS, Cambridge, UK) which were pretreated by mechanical and electrochemical polishing according to the following procedure. Before its first use, the electrode surface was briefly scoured with a silicon carbide emery paper of 1200 grit to obtain a fresh surface. To smoothen the resulting relatively rough surface, it was further subjected to sequential polishing by polishing cloth covered with alumina powder of 1, 0.3 and 0.05 mm particle size (Buehler, Lake Bluff, IL, USA) for, respectively, 5, 10 and 20 min. To remove any adherent Al_2_O_3_ particles, the electrode surface was rinsed thoroughly with doubly deionized water and cleaned in an ultrasonic bath containing deionized water (Branson 3210, FL, USA) for 2 min.

Before immobilizing the gelatin and Gel/CdS onto the electrode, the gold surface was modified with a self-assembled monolayer [42] of 6-mercaptohexanol (MH) (unless otherwise indicated). The latter was done by immersing the electrode in a water solution containing 1 mmolL^−1^ MH for 18 h at room temperature. The modified gold electrodes were consequently rinsed with water to remove any physically adsorbed MH. These modified electrodes were denoted as MH|Au. To immobilize gelatin and gelatin/CdS onto a MH|Au electrode or bare Au, 7 µL of corresponding solution (5, *w*/*v*%) was brought onto the surface by using a syringe and was exposed to air for 2 h at 4 °C (drop drying). The gelatin and Gel/CdS solution was prepared by mixing the corresponding film with HEPES buffer solution at 40 °C. These electrodes were referred to in the text as Au|Gel/CdS and Au|MOH|Gel/CdS. Finally, all electrodes were washed with the HEPES buffer solution and stored at 4 °C for further characterization. 

## Data Availability

The data presented in this study are available in article and supplementary material.

## Supplementary Materials

The following supporting information can be downloaded at: https://www.mdpi.com/article/10.3390/ijms231911867/s1.
