# Supplementary material for: Bio-Nanohybrid Gelatin/Quantum Dots for Cellular Imaging and Biosensing Applications
Source: Int J Mol Sci. 2022 Oct 6;23(19):11867. doi: 10.3390/ijms231911867 (PMC9570018; doi:10.3390/ijms231911867)
Supplement: Supplementary file 1 [file ijms-23-11867-s001.zip › ijms-1861866-supplementary.pdf]

Supporting Information

Bio-Nanohybrid Gelatin/Quantum Dots for Cellular Imaging and Biosensing Applications

Sangram Keshari Samal [1,2,3,*], Stefaan Soenen [4], Dario Puppi [5], Karolien De Wael [6], Sanghamitra Pati [1], Stefaan De Smedt [2], Kevin Braeckmans [2] and Peter Dubruel [3]

1 Laboratory of Biomaterials and Regenerative Medicine for Advanced Therapies, Indian Council of Medical Research-Regional Medical Research Center, Bhubaneswar 751013, Odisha, India
2 Laboratory of General Biochemistry and Physical Pharmacy, Center for Nano- and Biophotonics,
Ghent University, Ottergemsesteenweg 460, 9000 Ghent, Belgium
3 Polymer Chemistry & Biomaterials Research Group, Ghent University, Krijgslaan 281-S4, 9000 Gent, Belgium
4 Biomedical MRI Unit/MoSAIC, KU Leuven Department of Medicine, Herestraat 49, 3000 Leuven,
Belgium
5 BioLab Research Group, Department of Chemistry and Industrial Chemistry, University of Pisa, UdR INSTM-Pisa, Via Moruzzi 13, 56124 Pisa, Italy
6 Department of Chemistry, University of Antwerp, Universiteitsplein 1, 2610 Antwerp, Belgium
* Correspondence: sksamalrec@gmail.com

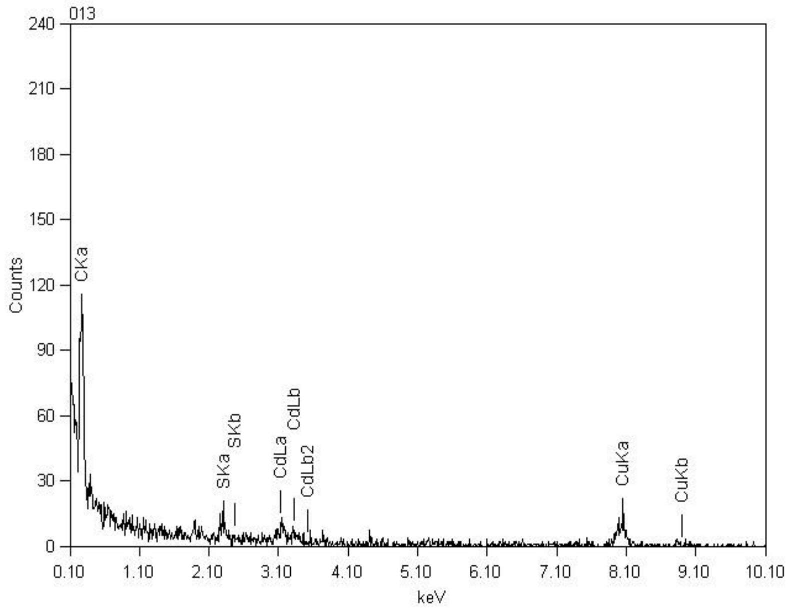

Figure S1. TEM-EDS spectra of Gel/CdS QdTs.

SEM-EDS analysis of Gelatin and Gel/CdS QDs:

The energy-dispersive spectroscopy (EDS) analysis was performed on a JEOL JSM-5600 instrument equipped with an electron microprobe JED 2300 and EDS detector for elemental analysis. The samples were coated with a thin conductive carbon layer by flash evaporation.

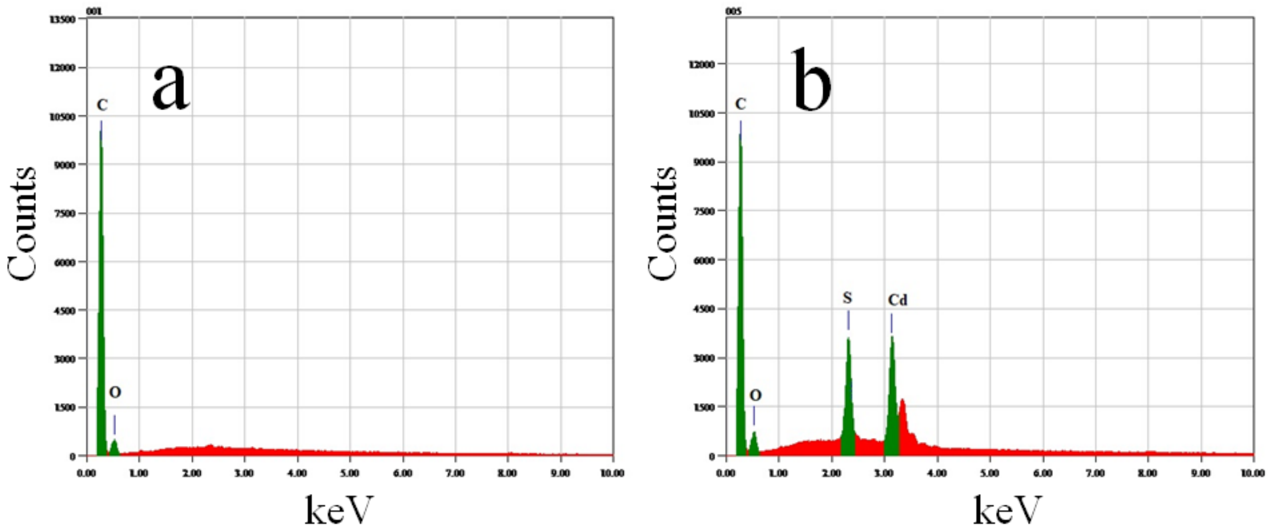

Figure S2. SEM-EDS spectra of Gelatin and Gel/CdS QdTs.
